# Dynamin inhibition causes context-dependent cell death of leukemia and lymphoma cells

**DOI:** 10.1371/journal.pone.0256708

**Published:** 2021-09-07

**Authors:** Christopher von Beek, Linnéa Alriksson, Josefine Palle, Ann-Marie Gustafson, Mirjana Grujic, Fabio Rabelo Melo, Mikael E. Sellin, Gunnar Pejler

**Affiliations:** 1 Department of Medical Biochemistry and Microbiology, Uppsala University, Uppsala, Sweden; 2 Department of Woman’s and Children’s Health, Uppsala University, Uppsala, Sweden; 3 Department of Medical Biochemistry and Microbiology, Science for Life Laboratory, Uppsala University, Uppsala, Sweden; European Institute of Oncology, ITALY

## Abstract

Current chemotherapy for treatment of pediatric acute leukemia, although generally successful, is still a matter of concern due to treatment resistance, relapses and life-long side effects for a subset of patients. Inhibition of dynamin, a GTPase involved in clathrin-mediated endocytosis and regulation of the cell cycle, has been proposed as a potential anti-cancer regimen, but the effects of dynamin inhibition on leukemia cells has not been extensively addressed. Here we adopted single cell and whole-population analysis by flow cytometry and live imaging, to assess the effect of dynamin inhibition (Dynasore, Dyngo-4a, MitMAB) on pediatric acute leukemia cell lines (CCRF-CEM and THP-1), human bone marrow biopsies from patients diagnosed with acute lymphoblastic leukemia (ALL), as well as in a model of lymphoma (EL4)-induced tumor growth in mice. All inhibitors suppressed proliferation and induced pronounced caspase-dependent apoptotic cell death in CCRF-CEM and THP-1 cell lines. However, the inhibitors showed no effect on bone marrow biopsies, and did not prevent EL4-induced tumor formation in mice. We conclude that dynamin inhibition affects highly proliferating human leukemia cells. These findings form a basis for evaluation of the potential, and constraints, of employing dynamin inhibition in treatment strategies against leukemia and other malignancies.

## Introduction

Acute leukemia is characterized by a rapid expansion of hematopoietic precursors (blast cells), which remain partially differentiated and proliferate in the bone marrow and peripheral blood as the disease progresses [1]. Two of the most common types of pediatric acute leukemias are acute lymphoblastic leukemia (ALL) and acute myeloid leukemia (AML). ALL is the most prevalent type of cancer in children (80% of leukemias and 25% of childhood cancers) [2, 3], and 10–15% of these cases represent T-cell ALL (T-ALL). T-ALL arises from T-lymphoblasts in the thymus as a result of an accumulation of mutations in immature thymocytes, altering *inter alia* cell proliferation and differentiation. The lymphoblasts leave the thymus and access the circulation, from where other organs such as the bone marrow or central nervous system are invaded [2]. AML constitutes about 20% of all childhood leukemias [4] and is caused by abnormal myeloblasts (immature leukocytes of the myeloid lineage). Cancerous myeloblasts have the ability of constant proliferation and avoidance of cell death, leading to an expansion in the myeloblast population and to a disturbed hematopoiesis [5].

Standard treatment of acute leukemia involves combination chemotherapy. Improved treatment strategies and reduced relapse has increased the 5-year survival to 90% [6]. However, in ALL, still 15% of the patients relapse [3] and AML has an overall survival rate of only 60–70% [7]. In addition, a major obstacle for the standard treatments are adverse side effects leading to long term organ damage associated with osteonecrosis, thrombosis, neurotoxicity, or chronic pancreatitis [8]. Therefore, new therapeutics with higher specificity are needed to increase the survival and quality of life for patients with childhood leukemia.

One potential chemotherapeutic strategy in leukemia would be to inhibit dynamin, a key component of the machinery for clathrin-mediated endocytosis and also for cytokinesis [9–11]. Indeed, this strategy has been evaluated previously, but only to a limited extent in leukemia and other cell types [12–16]. The most well-studied inhibitor of dynamin is Dynasore, which inhibits the GTP-hydrolase activity of dynamin [17]. Dynasore has been shown to affect multiple biological processes [18]. However, comparative studies employing interference with dynamin expression demonstrated that the observed effects might depend partially or completely on targets other than dynamin [19]. Due to other unfavorable properties of Dynasore, such as binding to serum proteins or loss of activity due to binding to detergents, multiple derivatives of Dynasore have been assessed. These efforts have resulted in the development of Dyngo-4a, which acts by a similar mechanism as Dynasore, but with a higher bioavailability [20]. Myristyl trimethyl ammonium bromide (MitMAB) is another, small molecule dynamin inhibitor, which reversibly targets the interaction of dynamin with phospholipids and thereby blocks the recruitment of dynamin to the membrane [21]. MitMAB was shown to inhibit dynamin’s GTPase-activity, and to inhibit receptor-mediated endocytosis in several cell types [22].

*In vivo* studies employing MitMAB are lacking to date. However, an *ex vivo* approach using rat kidneys did not result in visible symptoms of toxicity [23]. Chircop and colleagues performed a number of studies evaluating MitMAB as a potential anti-cancer agent. They reported that MitMAB inhibited the dynamin-dependent abscission in cytokinesis, leading to suppressed proliferation and polyploidy in a wide range of human cancer cell lines, while showing less toxicity for primary fibroblasts [24]. Dynamin inhibition is thereby emerging as a potential chemotherapeutic strategy, but its mechanism of action is not completely understood. Moreover, its effects on hematopoietic cancers have not been extensively evaluated to date. Here we addressed the effects of dynamin inhibition on leukemia cells, focusing on lymphoblastic and monocytic leukemia cells. Moreover, we addressed the mechanism through which dynamin acts on leukemia cells, and evaluated the *in vivo* toxicity and effect of dynamin inhibition in an EL4 lymphoma tumor model in mice.

## Materials and methods

### Cell lines and culture

We used CCRF-CEM (ATCC, CCL-19), a T-lymphoblast cell line derived from peripheral blood as model for ALL. THP-1 (ATCC, TIB-202) is a monocytic cell line representing AML. Cells were kept in RPMI-1640 (Sigma-Aldrich, #R5886), supplemented with 10% FBS (Gibco, #10500–064), 2 mM L-glutamine (Sigma-Aldrich, #G7513), 100 U/ml penicillin and 100 μg/ml streptomycin (Sigma-Aldrich, #P0781) in 5% CO_2_ at 37°C. For THP-1 cell culture, 50 μM 2-mercaptoethanol (Gibco, #31350–010) was added to the medium. Cell numbers were determined by trypan blue (Gibco, #15250–061) exclusion and quantified by an automated cell counter (Countess^™^ II FL; Life Technologies). EL4 (ATCC, TIB-39), a mouse T-lymphoblast leukemia cell line, was used for tumor generation in mice. EL4 cells were cultured as indicated for CCRF-CEM cells above, but at a 4 mM L-glutamine concentration in the medium. K562 (ATCC, CCL-243), a cell line representing chronic myelogenous leukemia, was cultured in RPMI-1640 with glutamax (Gibco, #72400) supplemented with 1 mM sodium pyruvate (Gibco, # 11360–070), FBS and streptomycin and penicillin as above. All centrifugations were performed at room temperature (RT) for 5 min, 300 x g.

### Human peripheral blood-derived mononuclear cells

Blood was obtained from healthy donors (Ethical permission: Etikprövningsmyndigheten, Dnr 2020–05080) after having obtained written consent. Mononuclear cells were purified with Percoll (Sigma-Aldrich, P1644) gradient centrifugation (30 min, 1000 x g, RT). Cells were washed 3 x in PBS (5 min, 300 x g, RT) and cell viability was determined by trypan blue staining. Mononuclear cells were used for experiments directly after isolation, and were kept in RPMI-1640 with Glutamax supplemented with sodium pyruvate, FBS and streptomycin and penicillin as described above for K562 cells.

### Human bone marrow cells

Bone marrow samples were obtained from ALL pediatric patients (Ethical permission: Etikprövningsmyndigheten, Dnr 2014/233; Uppsala Biobank application no BbA-827-2019-067), after having obtained written consents from parents/caretaker and from the child (where applicable). After thawing, bone marrow cells were washed and cultured in complete StemPro-34 SFM (Gibco, #10639011), including 2 mM L-glutamine, 100 U/ml Penicillin and 100 μg/ml Streptomycin. Viability was assessed by trypan blue exclusion. The cell concentrations were adjusted to 0.5 x 10^6^ cells/ml. Cells were treated with medium or MitMAB diluted in medium for 8 h and 24 h, followed by flow cytometry analysis.

### Mice and EL4 tumor model

C57BL/6J mice (8–16 weeks old) were used for experiments. All experiments were approved by the local ethics committee (Uppsala djurförsöksetiska nämnd, Dnr 5.8.18-04096/2019). Prior to injection, EL4 cells were resuspended in Hanks’ balanced salt solution (Sigma-Aldrich, #H6648) and the cell concentration was adjusted to 0.5 x 10^6^ cells/ml. A total of 5 x 10^4^ EL4 cells (100 μl of cell suspension) were injected subcutaneously in the hip region (both sides). From day 3 or 7 post-injection, intraperitoneal treatment with 5 or 10 mg/kg/day (3–4 times/week) MitMAB in PBS, or PBS only (vehicle) (100–200 μl per mouse) was initiated and the mice were examined for weight loss/gain. Tumor size was determined with a caliper (tumor volume = (a x b^2^)/2, with a: length, b: width). Mice were sacrificed when one tumor reached 1100 mm^3^.

### Viability assay

0.05–0.15 x 10^6^ cells/ml in 100 μl/well were transferred to 96-well plates (Sarstedt, #83.3924) and treated with Dyngo-4a (Selleck Chemicals, #8047), Dynasore monohydrate (Sigma-Aldrich, #D763) or MitMAB (Tocris, #4224). Where indicated, 20 μM of the pan-caspase inhibitor Z-VAD-FMK (BioVision, #1140) was added 1 h prior to treatment with dynamin inhibitors. As controls served the respective diluent (Cyclodextrin; Sigma-Aldrich, #C-4805) or PBS (Medicago, #009-9400-100, for MitMAB). At different time points, 10 μl of PrestoBlue (Invitrogen, #A13261) was added and the plate was incubated for 1–3 h before measurement of fluorescence on an Infinite M200 (TECAN). Fluorescence after blank subtraction (medium) were normalized to controls.

### Cell cycle analysis

1 ml of 0.15 x 10^6^ cells/ml suspensions of CCRF-CEM or THP-1 cells were treated with 40 μM Dynasore, 10 μM Dyngo-4a, 3 μM MitMAB, 10 μM RO-3306 (Sigma-Aldrich, # ML0569), 0.5 μg/ml Paclitaxel (Taxol, Sigma-Aldrich, T7402) and 2 μg/ml Cytochalasin D (Sigma-Aldrich, # C8273) or vehicle (DMSO for cell cycle inhibitors, otherwise as above) for 18 h before processing cells for flow cytometry.

### Transferrin uptake assay

CCRF-CEM and THP-1 cells were washed once in RPMI-1640 containing 1% BSA (Sigma-Aldrich, #A9418) and serum starved for 1 h and an additional 1 h after addition of 5 μM MitMAB in round bottom 96 well plates (Thermo Scientific, #163320); 200 μl/well, 0.15 x 10^6^ cells/ml. 25 μg/ml (final concentration) Transferrin-AlexaFluor 488 (Thermo Fisher Scientific, #T13342) was added for the indicated time periods, followed by washing in RPMI-1640 1% BSA and measurement of cell-associated fluorescence intensity by flow cytometry.

### Flow cytometry

For viability, cells were stained in annexin V-binding buffer (BD, #556454) with 0.5% annexin V-FITC (BD, #556419) and 150 nM DRAQ7 (Biostatus, #DR71000) prior to measurement by either a MACSQuant VYB (Miltenyibiotec), an Accuri C6 Plus (BD) or a Cytoflex S (Beckman Coulter). For proliferation, the Click-iT^™^ EdU Cell Proliferation Kit was used with Alexa Fluor 647 (Invitrogen, #C10424) according to the manufacturer’s protocol, but using half of the staining reagents in the click reaction step. 10 μM EdU was added 2 h prior to the assay. For caspase-3/7 detection, cells were resuspended in PBS containing 500 nM caspase-3/7 probe (Thermo Fisher Scientific, #C10423) and 5% FBS, and incubated for 30 min under standard culture conditions prior to flow cytometry analysis. Cells used for cell cycle analysis were resuspended in PBS containing 0.1% Triton-X-100 (Sigma-Aldrich, #T8787), 10 μg/ml RNase A (Thermo Fisher Scientific, #EN0531) and 10 μg/ml Propidium Iodide (Sigma-Aldrich, #81845) and incubated for 20–30 min in the dark at RT. For ALL bone marrow biopsies, cells were stained in annexin V-binding buffer containing 2% FBS, 0.5% annexin V-Pacific Blue (Thermo Fisher Scientific, #R37177) and 1 μM SYTOX Green (Thermo Fisher Scientific, #S7020). Data analysis was performed using the FlowJo software (TreeStar Inc.).

### Live microscopy

250 μl cell suspension (0.15 x 10^6^ cells/ml) in medium containing 150 nM DRAQ7 and 500 nM CellEvent^™^ Caspase-3/7 were added to 8-well chamber slides (Nunc^®^ Lab-Tek^®^ II—CC^2™^ Chamber Slide^™^; ThermoFisher Scientific). Slides were incubated at 37°C (5% CO_2_) during microscopy and equilibrated in the microscope chamber 1 h prior to the start of the experiment. Images were acquired on a custom-built microscope, based on a Nikon Ti2 body, every 10 min in differential interference contrast (DIC), red and green fluorescence channels with a Prime 95B (Photometrics) camera through a 10X/0.45 NA Plan Apochromatic objective (Nikon). Micro Manager was used for controlling the microscope (μManager plugin [25] and Fiji [26] were used for analyzing images).

### Statistical analysis

Data are presented as mean values + standard error of the mean (SEM) if not otherwise indicated. Descriptions of the statistical tests used in each case can be found in the figure legends. Microsoft Excel 2016 and GraphPad Prism 9 were used to analyze and plot the data. Significance levels: *p < 0.05; **p < 0.01; ***p < 0.001.

## Results

### Dynamin inhibitors potently decrease survival of acute leukemia cell lines

**Fig 1:**
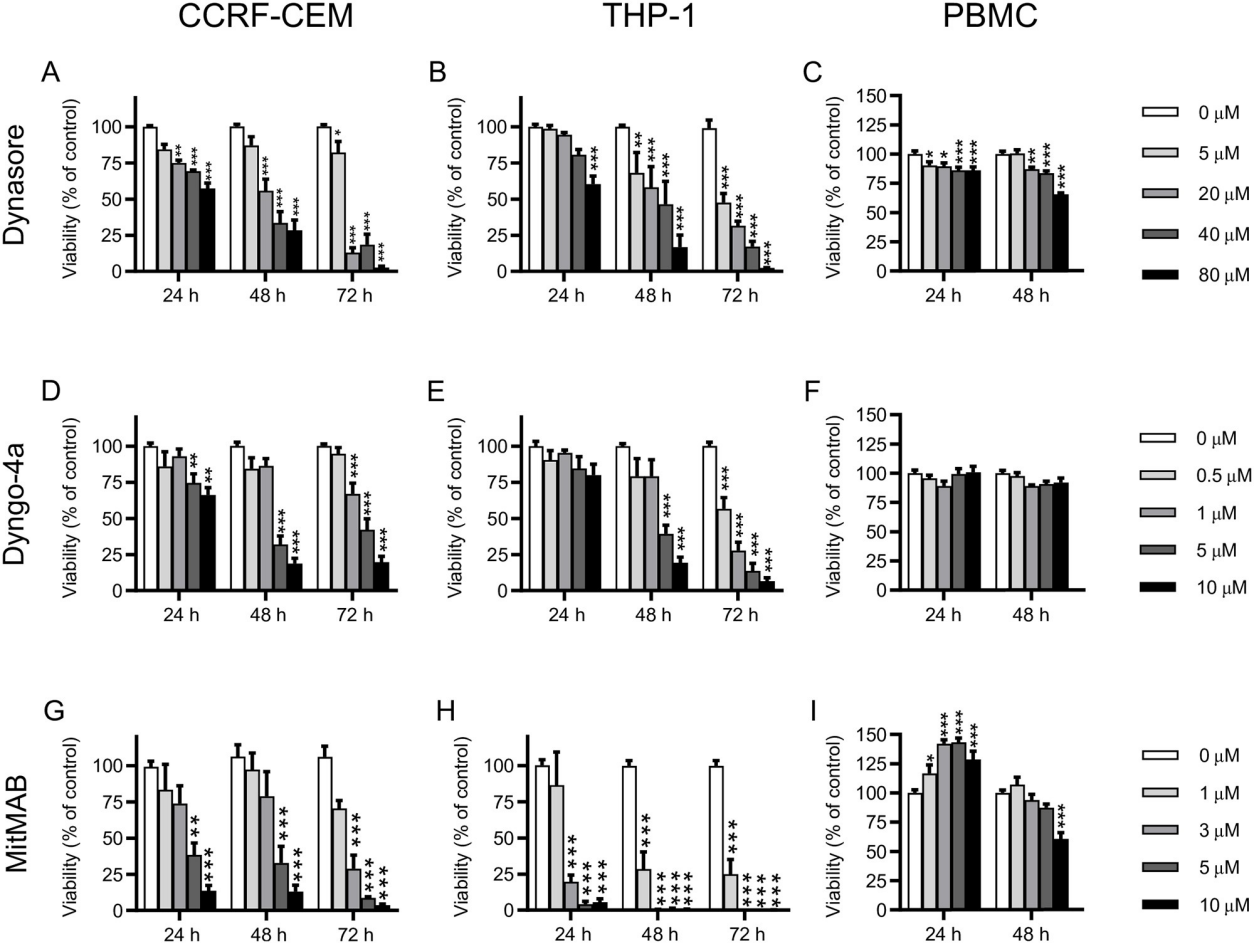
Dynamin inhibitors decrease the viability of acute leukemia cells. CCRF-CEM (A, D, G;), THP-1 (B, E, H) or human PBMCs (C, F, I) (0.15 x 10^6^ cells/ml) were cultured for the time points indicated with the given concentrations of Dynasore (A-C), Dyngo-4a (D-F) or MitMAB (G-I). Viability was assessed with PrestoBlue and was normalized to untreated control (0 μM) groups. Data are given as mean + SEM pooled from 5–9 biological replicates. Two-way ANOVA was used for statistical testing with Dunnett’s posthoc test in order to compare each treated group with the control at each time point.

First, we investigated the effects of three different dynamin inhibitors on the CCRF-CEM and THP-1 cell lines, as models for pediatric ALL and AML, respectively. All inhibitors induced a prominent reduction in viability in both cell lines (Fig 1A, 1B, 1D, 1E, 1G and 1H). The effects of all inhibitors showed time- and concentration-dependency, and the effects lasted at least up to 72 h. Among the assessed inhibitors, MitMAB was most efficient, followed by Dyngo-4a and Dynasore. It was also noted that THP-1 cells were relatively resistant to low concentrations of Dynasore and Dyngo-4a (Fig 1B and 1E), whereas MitMAB showed a high efficacy on THP-1 cells also at low concentrations (Fig 1H). In addition, we found that K562 cells, representing a myelogenous leukemia cell line, were susceptible to dynamin inhibition, although with somewhat lower sensitivity in comparison with the THP-1 and CCRF-CEM cells (S1 Fig).

We also assessed whether dynamin inhibition had the capacity to suppress peripheral blood-derived mononuclear cells (PBMCs), a population comprising both monocytes and lymphocytes. However, neither of the dynamin inhibitors had any substantial cytotoxic effects on these cells after a 24 h incubation period (Fig 1C, 1F and 1I). Notably, one of the dynamin inhibitors (MitMAB) appeared to cause a slightly enhanced cell survival at early time points, while such effects were not seen for the other two assessed dynamin inhibitors. After 48 h, a limited reduction in viability was seen at the higher concentrations of Dynasore and MitMAB (Fig 1C and 1I). This suggests that dynamin inhibition has a more pronounced inhibitory effect on leukemia cell lines than on healthy PBMCs kept in culture.

### Dynamin inhibitors induce leukemia cell apoptosis

**Fig 2:**
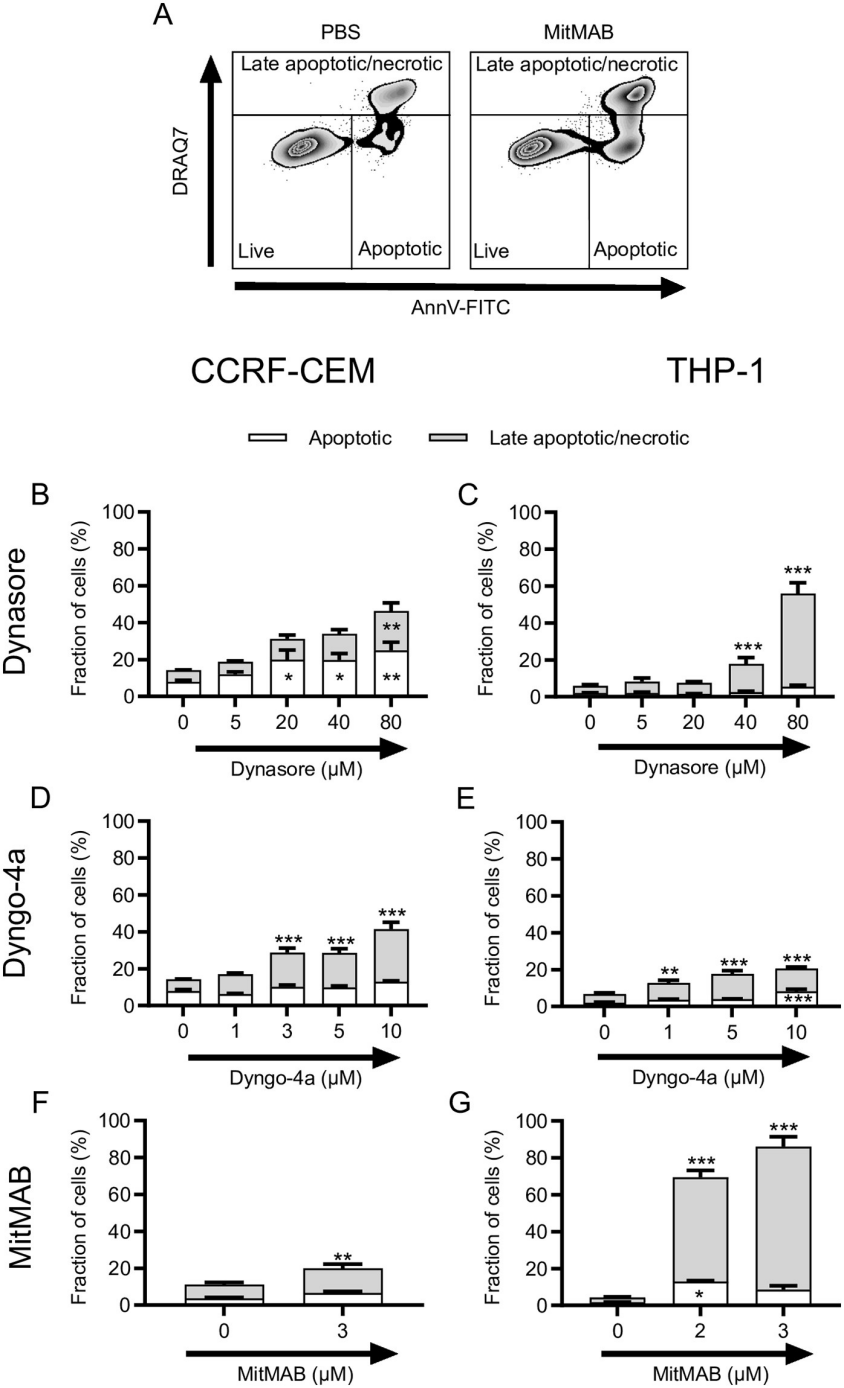
Dynamin inhibition leads to apoptosis of leukemia cells. (A) Gating strategy. (B-G) 0.15 x 10^6^ CCRF-CEM (B, D, F) or THP-1 (C, E, G) cells/ml were cultured for 24 h with the indicated concentrations of Dynasore (B-C), Dyngo-4a (D-E) or MitMAB (F-G), followed by staining with Annexin V and DRAQ7. Percentage of viable (Annexin V^-^/DRAQ7^-^), apoptotic (Annexin V^+^/DRAQ7^-^) and late apoptotic/necrotic (Annexin V^+^/DRAQ7^+^) was determined by flow cytometry. Data are given as mean + SEM, pooled from at least 6 biological replicates. Two-way ANOVA was used for statistical testing with Dunnett’s posthoc test (Sidak for MitMAB experiments due to fewer groups), comparing the treated groups with the controls.

To gain insight into the mechanism of viability loss induced by the dynamin inhibition, we performed staining with annexin V (binds to phosphatidylserine) and DRAQ7 (nuclear dye that enters permeabilized cells) (Fig 2; gating strategy shown in Fig 2A). As seen in Fig 2B, treatment of CCRF-CEM cells with Dynasore for 24 h resulted in an increased proportion of early apoptotic cells (annexin V^+^/DRAQ7^-^), although late apoptotic/necrotic (annexin V^+^/DRAQ7^+^) cells were also observed. After treatment of the CCRF-CEM cells with Dyngo-4a or MitMAB for a similar time period, early apoptotic cells were again detected, but a higher proportion of late apoptotic/necrotic cells were observed in comparison with the Dynasore treatment (Fig 2D and 2F). A significant increase in the proportion of early apoptotic cells was also seen after treatment of THP-1 cells with the dynamin inhibitors (Fig 2C, 2E and 2G). However, treatment of THP-1 cells with high concentrations of Dynasore, or with modest concentrations of MitMAB, resulted in a large proportion of late apoptotic/necrotic cells (Fig 2C, 2E and 2G). Nevertheless, these data suggest that dynamin inhibition elicits apoptosis in leukemia cell lines.

### Dynamin inhibitors reduce the proliferation of leukemia cells

**Fig 3:**
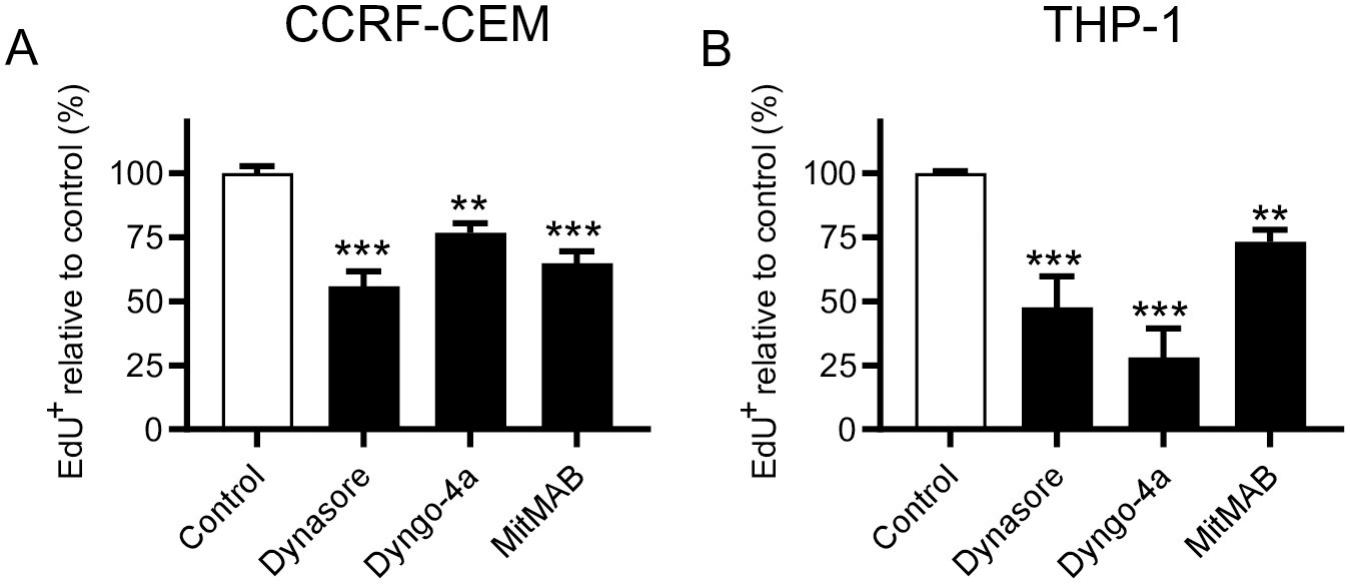
Dynamin inhibition reduces the proliferation of leukemia cells. 0.15 x 10^6^ of CCRF-CEM (A) or THP-1 (B) cells/ml were cultured for 24 h (MitMAB) or 48 h (Dynasore and Dyngo-4a) with 40 μM Dynasore, 10 μM Dyngo-4a, or 2 μM MitMAB (3 μM for CCRF-CEM). EdU was added to a final concentration of 10 μM 2 h prior to flow cytometry analysis. The proportion of proliferating (EdU^+^) cells was normalized to untreated cells. The raw fraction of proliferating cells in CCRF-CEM was 52.64 ± 6.48% (mean±standard deviation) and 40.56 ± 13.38% for THP-1. Data are given as mean + SEM, pooled from 6 biological replicates. One-way ANOVA was used for statistical testing with Dunnett’s posthoc test.

Next, we investigated whether dynamin inhibition affected the proliferative capacity of the leukemia cells. For this, we labeled cells with EdU, a thymidine analogue used for quantification of DNA synthesis, and measured EdU incorporation by flow cytometry. As seen in Fig 3A and 3B, all of the dynamin inhibitors caused a significant reduction in the proliferation of both CCRF-CEM and THP-1 cells. Notably, THP-1 cells were somewhat more sensitive than were the CCRF-CEM cells.

### MitMAB-induced cell death involves caspase-3/7 activation

**Fig 4:**
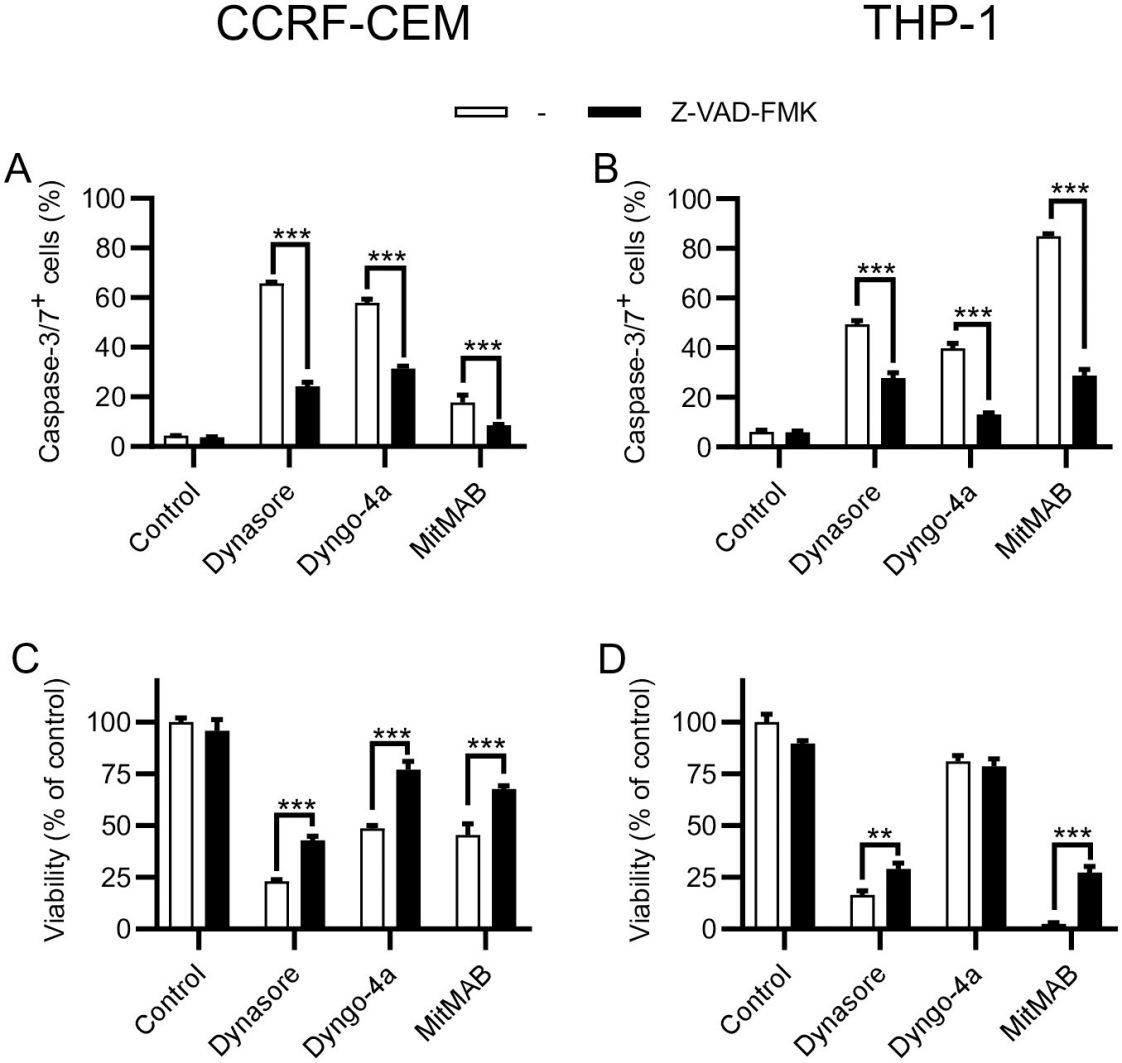
Inhibition of dynamin leads to caspase-3/7-dependent apoptosis in leukemia cells. 0.15 x 10^6^ of CCRF-CEM (A, C) or THP-1 (B, D) cells/ml were cultured for 24 h with 40 μM Dynasore, 10 μM Dyngo-4a or 3 μM MitMAB in the absence or presence of 20 μM Z-VAD-FMK (pan-caspase inhibitor) as indicated. (A-B) Caspase activity was analyzed by flow cytometry after addition of a caspase-3/7 probe. (C-D) Cell viability was assessed with PrestoBlue; treated groups were normalized to control groups. Data are given as mean + SEM, containing 4 pooled replicates from two independent experiments. Two-way ANOVA was used for statistical testing with Sidak’s posthoc test.

A hallmark feature of apoptotic cell death is the generation of active caspase-3/7. Since our data suggest that leukemia cells subjected to dynamin inhibition show signs of apoptosis, we assessed whether this is accompanied by caspase-3/7 activation. These experiments revealed a robust activation of caspase-3/7 in response to all dynamin inhibitors, both in the CCRF-CEM and THP-1 cells (Fig 4A and 4B). In the presence of Z-VAD-FMK, a pan-caspase inhibitor, caspase-3/7 activities were significantly reduced (Fig 4A and 4B). To assess the functional impact of caspase activation upon dynamin inhibition, we next analyzed leukemia cell survival. As depicted in Fig 4C and 4D, caspase inhibition by Z-VAD-FMK caused a significant, although not complete, restoration of cell viability in dynamin-inhibited leukemia cells. The only exception to this was seen after treatment of THP-1 cells with Dyngo-4a, where only a modest impact on cell viability was observed (Fig 4D, see also Fig 1D), and where no protective effective of Z-VAD-FMK was observed within 24 h (Fig 4D).

### Caspase-3/7 activation precedes cell death in dynamin-inhibited leukemia cells

**Fig 5:**
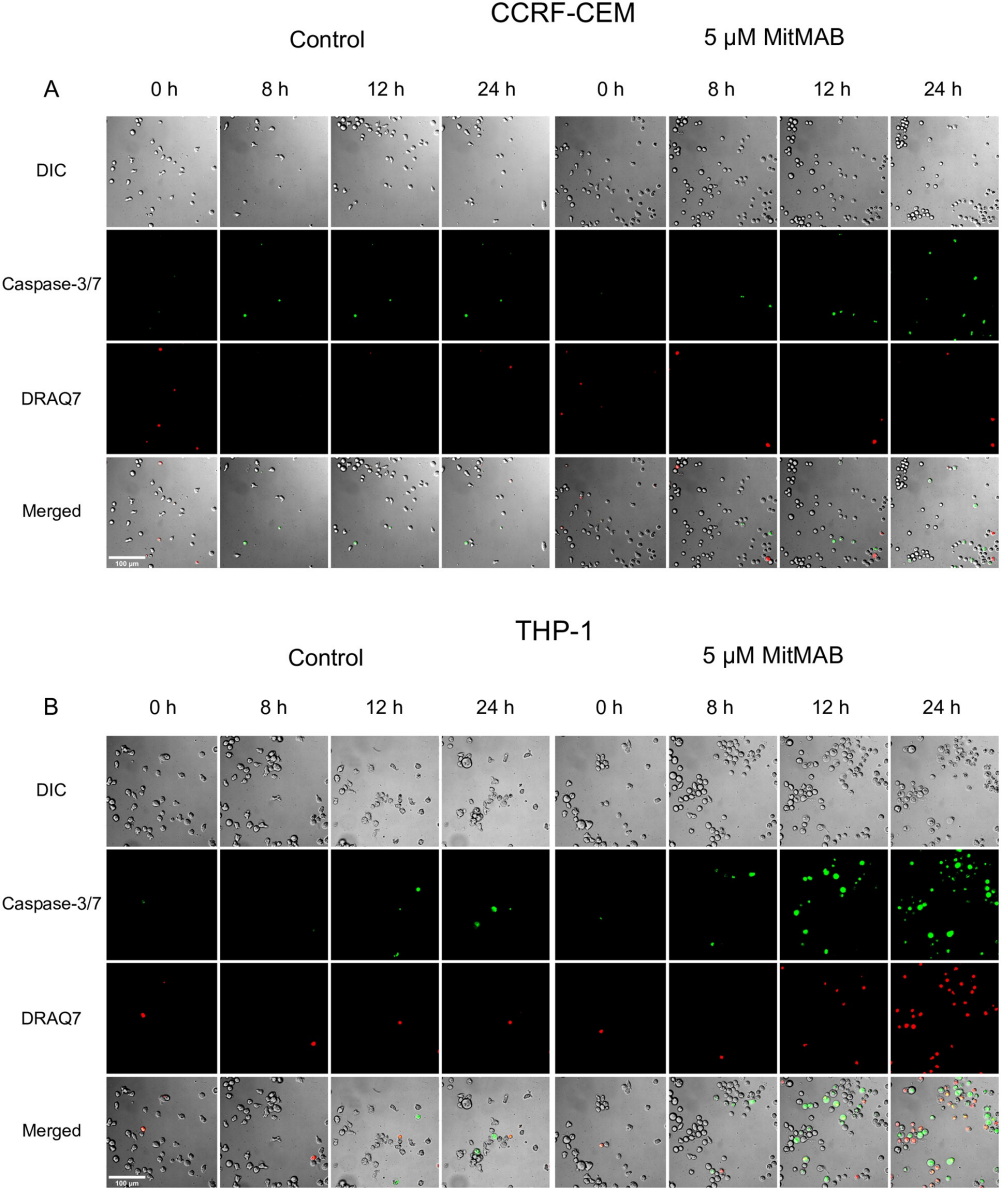
Caspase activation precedes overt cell death in leukemia cells subjected to dynamin inhibition. 0.15 x 10^6^ of CCRF-CEM (A) or THP-1 (B) cells/ml in medium containing 150 nM DRAQ7 and 500 nM caspase-3/7 probe were added to chamber slides, followed by addition of MitMAB (5 μM final concentration) as indicated. Every 10 min, images of every well were taken. The indicated DIC, fluorescence, or merged channels are shown. Scale bars = 100 μm. Representative images of two wells per condition are shown (see also S1–S4 Movies).

To provide further insight into the dynamics by which dynamin inhibition triggers cell death in leukemia cells, we used live microscopy, in which we monitored caspase-3/7 activation and DRAQ7 staining (i.e., cells with a permeabilized plasma membrane) in real time after addition of 5 μM MitMAB. In line with results above (Figs 1G, 1H, 2F and 2G), MitMAB elicited modest caspase-3/7 activation in CCRF-CEM cells within the first 24 h, whereas pronounced caspase-3/7 activation was seen in THP-1 cells (Fig 5A and 5B). This analysis further revealed that, in CCRF-CEM cells, caspase-3/7 activation was seen starting from ~8–12 h, and overt cell death (as judged by DRAQ7 positivity) was observed from 24 h and onwards (Fig 5A and S1 and S2 Movies). In THP-1 cells, the first signs of cell death were seen from ~8 h (Fig 5B and S3 and S4 Movies). Also here, individual dynamin-inhibited cells first turned caspase-3/7-positive, and thereafter DRAQ7-positive (S4 Movie).

### Dynamin-induced cell toxicity is independent of cell cycle inhibition, but interferes with transferrin uptake

**Fig 6:**
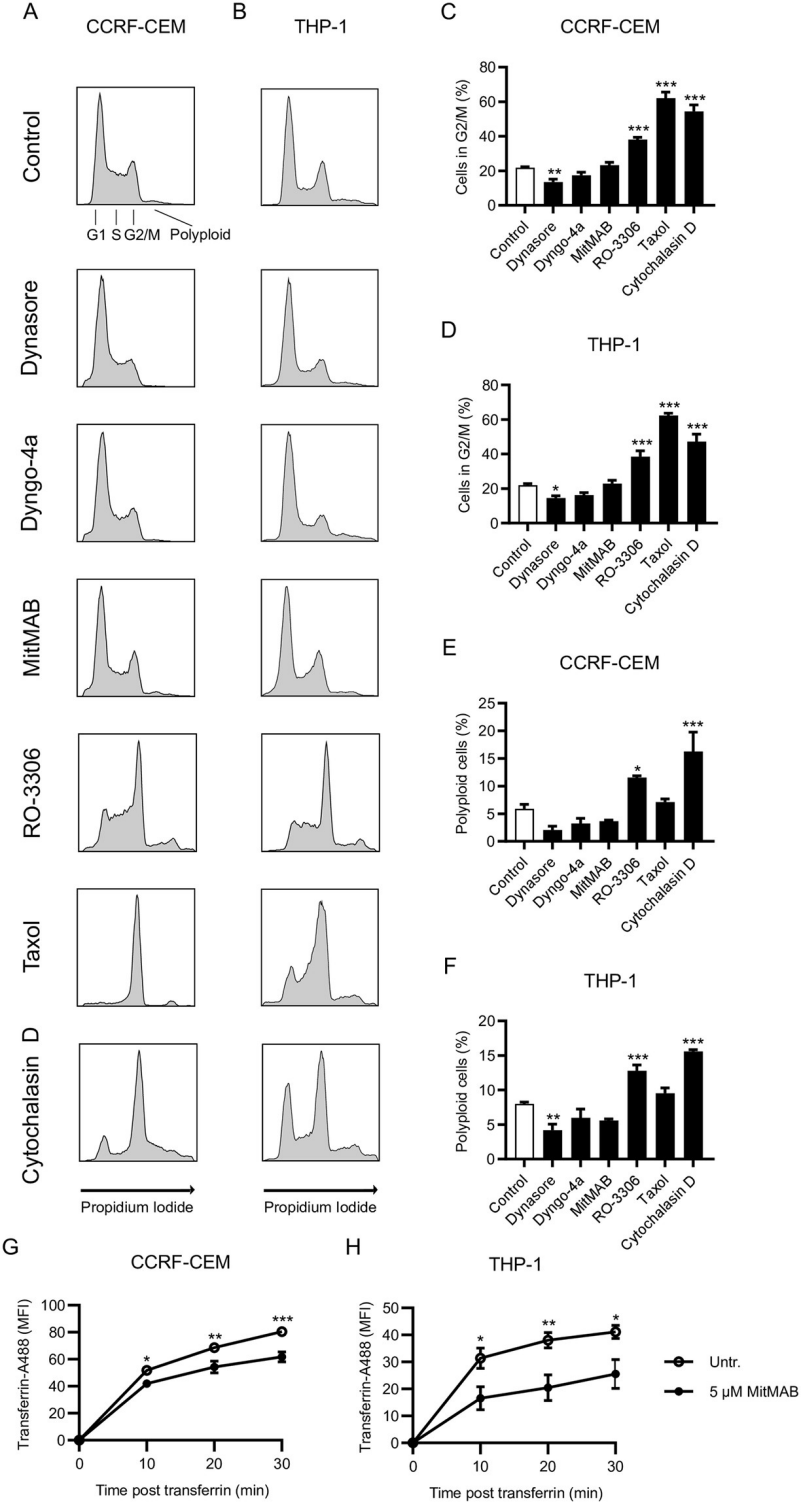
Dynamin inhibition affects transferrin uptake but not cell cycle progression in leukemia cells. (A-B) 0.15 x 10^6^ CCRF-CEM (A) or THP-1 (B) cells/ml were cultured for 18 h with either 40 μM Dynasore, 10 μM Dyngo-4a, 5 μM MitMAB, 10 μM RO-3306, 0.5 μg/ml Taxol, or 2 μg/ml Cytochalasin D as indicated. Cell cycle profiles were analyzed (see experimental procedures for details). Gating for different cell cycle phases: G1 (gap phase 1), S (DNA synthesis phase), G2/M (gap phase 2 or mitosis), as well as polyploid cells are shown in the upper left panel in (A). (C-F) Quantification of the proportion of cells in G2/M phase and polyploid cells as indicated. (G-H) Effect of MitMAB (5 μM) on the uptake of fluorescent transferrin by CCRF-CEM (G) and THP-1 (H) cells. Time point “0” indicates unstained samples. Data are given as mean + SEM, containing 4–6 pooled replicates from 2–3 independent experiments. Two-way ANOVA was used for statistical testing including Dunnett’s posthoc test for (D-F).

In the next set of experiments we investigated whether dynamin inhibition in leukemia cells had an impact on the regulation of a specific cell cycle stage, in analogy with previous studies in HeLa and H460 cells where dynamin inhibition was proposed to target cytokinesis [27]. However, the inhibition of dynamin by Dyngo-4a or MitMAB did not alter the distribution of cells in the various cell cycle phases, nor did it result in an increased population of polyploid cells, either in CCRF-CEM or in THP-1 cells (Fig 6A–6F). Dynasore even produced a small, but statistically significant, reduction of cells in G2/M, both in CCRF-CEM and THP-1 cells (Fig 6A–6F). As positive controls, we assessed the effects of the cdk1 inhibitor RO-3306 (arrests the cell cycle in the G2/M transition), paclitaxel (Taxol; arrests cells in metaphase) and cytochalasin D (arrests cells in the cytokinesis phase) [28–30]. As seen in Fig 6A–6F, all of these compounds caused a profound reduction in the proportion of cells in G1 phase, accompanied by a corresponding increase in the G2/M population. As expected, these established cell cycle inhibitors, in particular cytochalasin D, caused an increase in the proportion of polyploid cells, in line with defective cytokinesis. By sharp contrast, neither of the dynamin inhibitors caused an increase in the population of polyploid cells (Fig 6E and 6F).

Inhibition of endocytosis provides the other plausible mechanism for the cytotoxicity of dynamin inhibitors. We therefore explored whether MitMAB affects endocytosis in the leukemia cells, as determined by uptake of fluorescent transferrin. Indeed, MitMAB had a significant inhibitory effect on transferrin uptake in both CCRF-CEM and THP-1 cells (Fig 6G and 6H). Notably, MitMAB had a more profound inhibitory impact on transferrin endocytosis in the THP-1 cells in comparison with its effects on the CCRF-CEM cells (Fig 6G and 6H), which agrees well with the especially strong inhibitory effect of MitMAB on THP-1 viability (Figs 1G, 1H, 4C and 4D).

### Dynamin inhibition does not affect bone marrow cells from pediatric leukemia subjects

**Fig 7:**
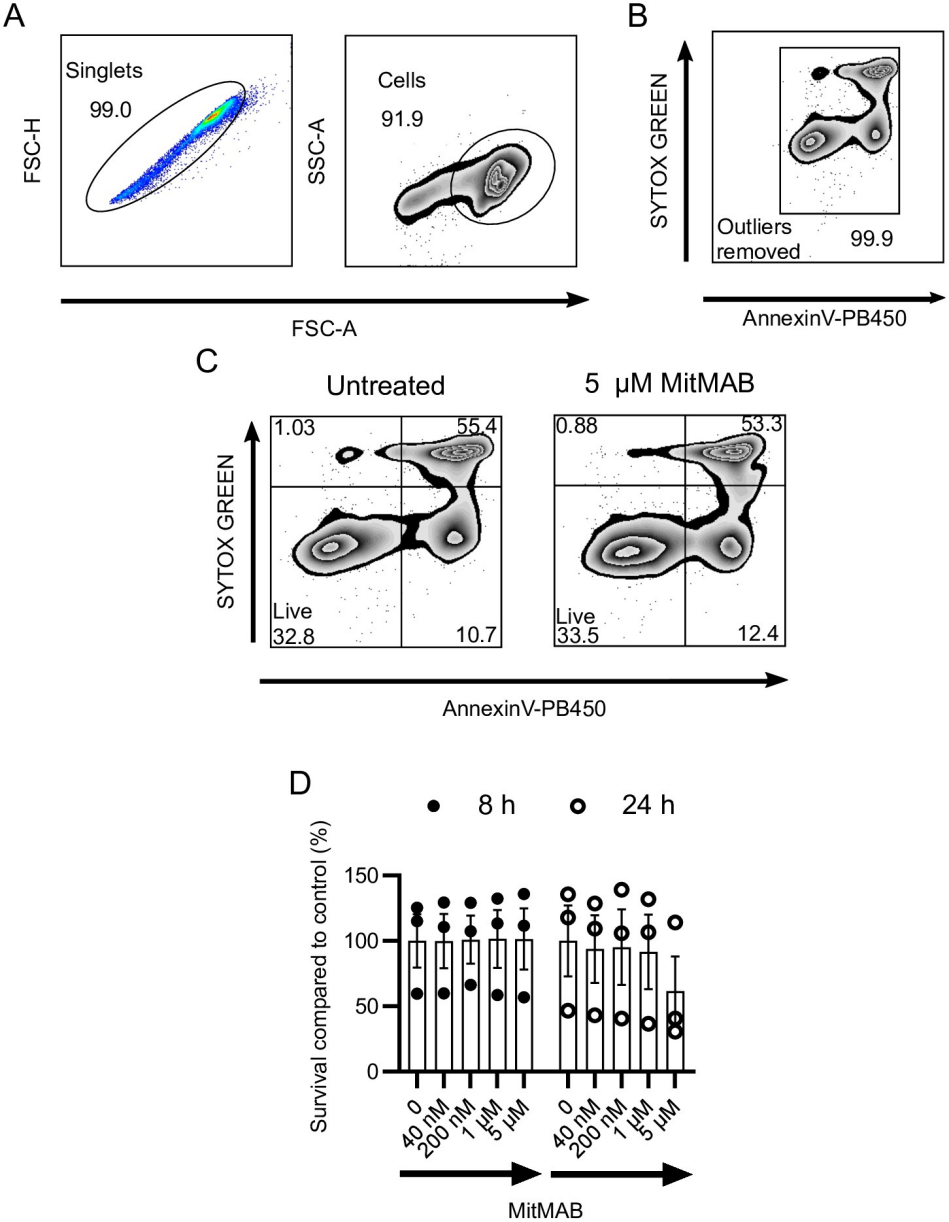
Dynamin inhibition does not affect the viability of pediatric leukemia bone marrow cells placed in liquid culture. (A-C) General gating strategies consisting of removal of duplets and debris (A), removal of outliers in fluorescence channels (B) and differentiation of populations regarding viability (C). (D) 0.5 x 10^6^ bone marrow cells from ALL patients were treated with the indicated concentrations of MitMAB for 8 h or 24 h and stained with Annexin V and SYTOX Green. The data shown represent mean values ±SEM (n = 3, data points indicate individual patients). Two-way ANOVA was used for statistical testing (no significant differences noted).

To estimate if the observed effects in leukemia cell lines translate into patient material, we utilized bone marrow samples from children diagnosed with ALL. For these experiments we assessed the effects of MitMAB, based on its superior potency in comparison with Dynasore and Dyngo-4a on CCRF-CEM and THP-1 cells (see Fig 1). However, MitMAB did not cause reduced viability/increased amount of apoptotic or late apoptotic cells, except for a tendency at the highest concentration (5 μM) over 24 h (Fig 7A–7D). It should be noted that non-culture-adapted bone marrow samples initially grow slowly in culture (compared to the adapted cell lines) and exhibit a comparatively high level of spontaneous cell death. Hence, these results may suggest that dynamin inhibition preferentially affects leukemia cells under conditions of fast growth.

### Dynamin inhibition does not affect tumor growth in a mouse lymphoma model

**Fig 8:**
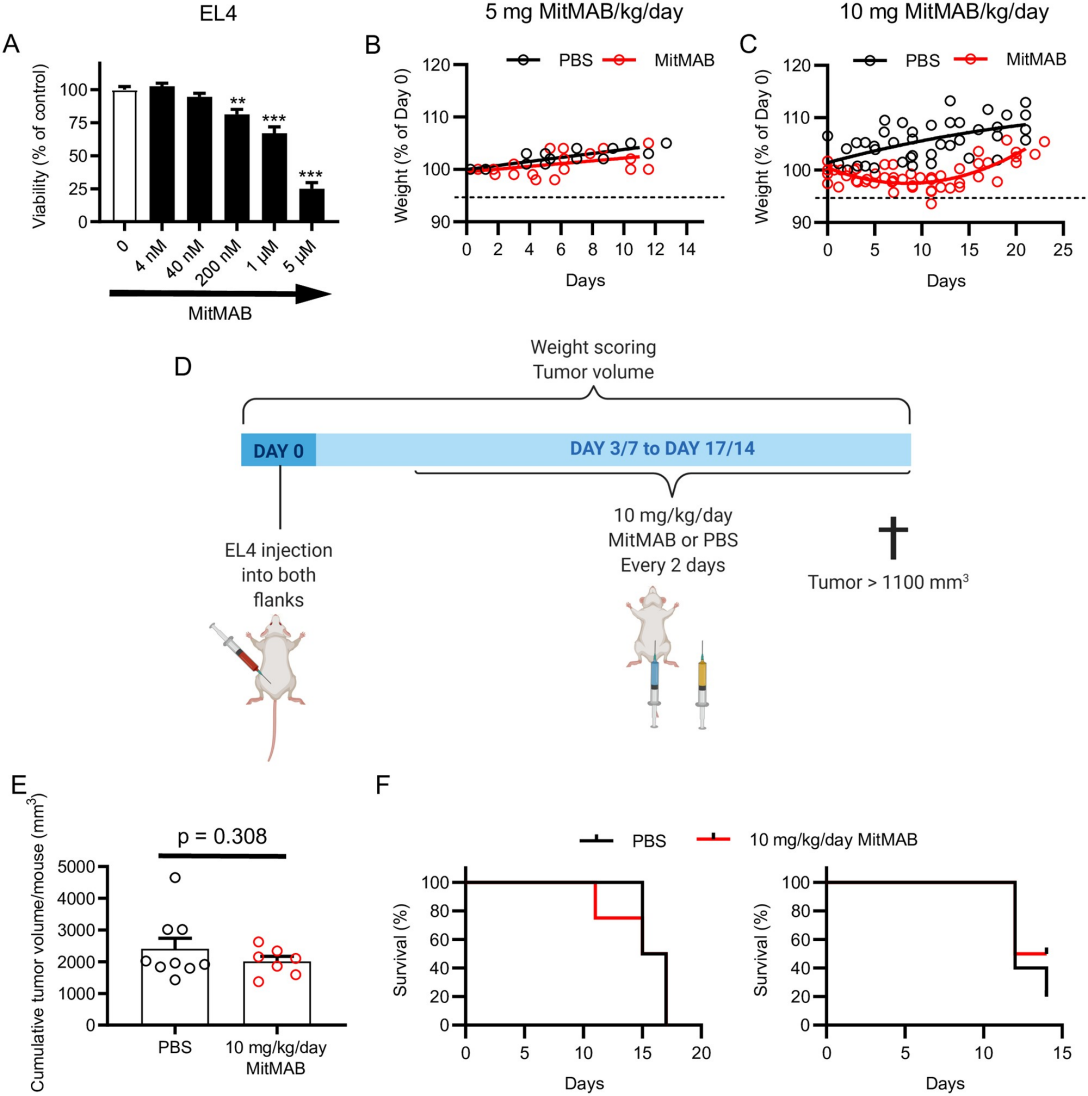
Dynamin inhibition does not prevent EL4 tumor growth in mice. (A) 0.15 x 10^6^ EL4 cells were treated with the indicated concentrations of MitMAB for 24 h, followed by cell viability measurement using PrestoBlue (n = 8; data pooled from two independent experiments). Groups were compared to control with one-way ANOVA and Dunnet’s posthoc tests. (B-C) Mice were injected intraperitoneally with 5 (n = 4) or 10 mg/kg/day (n = 4–5) MitMAB in PBS or PBS alone. Weight is shown relative to the treatment start and the dashed line indicates the ethical end point (5% weight loss). In (B), linear regression was used (p = 0.387); in (C) nonlinear fits are shown (two way ANOVA for treatment factor p < 0.0001%). (D-F) Mice were injected subcutaneosly with 5 x 10^5^ EL4 cells. After 3 or 7 days, treatment with MitMAB (10 mg/kg/day) was initiated (weight loss of 10% as ethical endpoint in this experiment). (E) Pooled tumor volume endpoints from two independent experiments. Every point shows the total tumor burden in a mouse (from both flanks). The groups were compared with Welch’s two-tailed t-test, n = 7–9. (F) Survival curves for the two experiments, with MitMAB treatment initiated on either day 3 (left graph), or day 7 (right graph).

Next, we investigated whether dynamin inhibition has the capacity to reduce tumor growth in a mouse model. For this we used a model utilizing subcutaneous injection of EL4 lymphoma cells into mice. In this setting we again used MitMAB, based on its observed more potent effects on leukemia cells in comparison with other tested dynamin inhibitors (see Fig 1). First, we conducted experiments to assess whether MitMAB was cytotoxic for the suspension-growing mouse lymphoma cells. Indeed, MitMAB caused reduced viability of the EL4 cells, with similar potency as for the CCRF-CEM and THP-1 cells (Fig 8A). To assess the general tolerability of mice for MitMAB, the compound was injected at 5 or 10 mg/kg/day (3 times/week). These experiments revealed that MitMAB at 5 mg/kg/day had minimal impact on the behavior of the animals, and no adverse effects on weight were seen (Fig 8B). However, at 10 mg/kg/day, MitMAB caused reduction of body weight at early time points after initiating treatment. This is an indication that the drug was taken up and had systemic effects. The body weights of the animals partially recovered after 20 days of treatment (Fig 8C). Nevertheless, this analysis defined the upper boundary for MitMAB administration *in vivo*. Based on these assessments, the effect of MitMAB at 10 mg/kg/day on tumor growth was tested (experimental setup shown in Fig 8D). However, as seen in Fig 8E and 8F, no detectable effect of dynamin inhibition on the subcutaneous tumor growth or animal survival was seen under these conditions. Hence, MitMAB has a potent, but highly context-dependent, cytotoxic effect on leukemia and lymphoma cells.

## Discussion

In this study we investigated the effects of dynamin inhibition on leukemia and lymphoma cells. Our results show that dynamin inhibition is highly cytotoxic to CCRF-CEM and THP-1 pediatric leukemia cells, which represent rapidly growing cells. In contrast, we observed no inhibiting effect on the viability of bone marrow cells from leukemia patients. However, these thawed bone marrow cells exhibit a comparatively high degree of spontaneous cell death, and are largely non-proliferating and thereby metabolically relatively inactive. Hence, we favor the notion that dynamin inhibition preferentially impacts on proliferating cells. Notably though, previous investigations have suggested that dynamin inhibition can have an impact on cells with both low and high proliferative capacity [16]. Nevertheless, we here provide data on the effect of dynamin inhibition on non-adherent cancer cells, which complements previous studies where the focus has predominantly been on adherent cell lines [12, 13, 24, 31]. Our findings reveal that dynamin inhibition induces potent caspase-dependent cell death through classical apoptosis.

Previous studies have revealed a major function for dynamin in two cellular processes—endocytosis and during the abscission step in cytokinesis [11]. It is thus possible that the effect of dynamin inhibition on cancer cells can be explained by interference with either or both of these processes. Of these possible scenarios, previous studies have mainly proposed that the effects of dynamin inhibition on cancer cells are explained by interference with cytokinesis [12, 13, 24, 27]. However, in this study we did not see any effect of dynamin inhibition on the progression through a specific cell cycle phase or checkpoint. This favors the notion that the cytotoxic effects on leukemia cells are independent of interference with cytokinesis, or any other discrete cell cycle event. In further agreement with this notion, dynamin inhibition caused only a partial blockade of EdU incorporation, suggesting minor effects on DNA synthesis. Possibly, these differences between the current study and earlier reports may lie in the nature of the studied cells, i.e., that our study focused on non-adherent leukemia cells whereas previous studies focused predominantly on adherent, mostly epithelial-derived cancer cells. In addition, in hepatocellular carcinoma cells, Dynasore, but not Dyngo-4a, was shown to affect the cell cycle distribution [31], which points to cell type-dependent effects of at least some of the dynamin inhibitors. Although we did not see any effects of dynamin inhibition on the cell cycle progression, using either MitMAB or Dyngo-4a at concentrations that effectively induce cell death of the leukemia cells, we cannot exclude that effects on the cell cycle (e.g., cytokinesis) could be seen at higher concentrations. Of note, we found that Dynasore, which was used at higher concentrations than both Dyngo-4a and MitMAB (to induce robust cell death), in fact caused a significant decrease of cells in the G2/M phase. This may hint towards a partial block elsewhere in the cell cycle, but this effect was subtle (<2-fold), even at the high concentrations of dynamin inhibitor used.

Based on the reasoning above, we propose that the cytotoxic effect of dynamin inhibition on leukemia cells is explained by interference with endocytosis, rather than with cell cycle control. In this context, it is notable that observed effects on cell cycle progression in cultured cells do not necessarily reflect the mechanism of action in humans. An important example of the latter is the current controversy about the mechanism of action for paclitaxel in human patients, for which its efficacy as a treatment regimen might be less related to cell cycle inhibition than originally proposed [32].

Here we show that MitMAB interferes with transferrin uptake, possibly leading to a shortage of iron. However, it is noteworthy that MitMAB at a concentration that profoundly impacted on cell viability (5 μM) caused only partial inhibition of transferrin uptake. This points to the involvement of also other endocytosis-related, or non-related, mechanisms in the induction of the cell death response. In line with this, it has recently been suggested that dynamin inhibition blocks ligand-bound receptor internalization in leukemia cells, leading to apoptosis [16].

Overall, it is reasonable to assume that highly proliferating cells such as the CCRF-CEM and THP-1 cell lines have a high demand for endocytic processes, which could serve to supply the cells with nutrients or have a function in essential recycling/turnover of cell surface molecules such as various receptors. According to this scenario, it would be expected that relatively quiescent cells, such as the bone marrow cells used in this study, are resistant to the effects of dynamin inhibition, which indeed is in agreement with our findings. On the other hand, it cannot be excluded that the bone marrow samples contain a limited fraction of transformed cells, and that effects of MitMAB on transformed cell populations may to some extent have been masked by non-responding (non-malignant) cells present in the bone marrow aspirates. In further support for this scenario, we noted that human peripheral blood-derived mononuclear cells were relatively unresponsive to dynamin inhibition.

We also assessed whether dynamin inhibition could have an impact on tumor growth in a mouse model based on subcutaneous injections of suspension-growing EL4 lymphoma cells. However, the treatment did not prevent or delay the tumor growth, and did not alter the survival of the treated mice vs. controls. The dynamin inhibitor used was well tolerated by the mice. Several reasons for the lack of efficacy of dynamin inhibition in this model can be envisioned. For example, we cannot be certain as to whether the dynamin inhibitor used is bioavailable at the site of tumor growth, and whether the drug actually penetrates into the tumor mass [33, 34]. Furthermore, it remains possible that MitMAB partially or completely loses biological activity in the *in vivo* milieu. It should also be emphasized that we used a previously established subcutaneous approach in this study, whereas a model based on intravenous administration of lymphoma cells might more closely reflect a setting of leukemia. Clearly, further in-depth investigations including pharmacokinetic assessments are thus needed to fully evaluate the possibility of using different dynamin inhibitors to dampen tumor growth across various *in vivo* models of leukemia and lymphoma (see also [16]). Interestingly, it was previously shown that mutations in the gene for dynamin II (DNM2), are frequent in ALL patients, hence providing support for a role of dynamin-dependent mechanisms in leukemia development [35].

In summary, this study provides important insights into how dynamin inhibition can potentially be exploited for anti-leukemia purposes, but also indicates key challenges that may stand in the way of clinical translation.

## Supporting information

S1 Fig Dynamin inhibitors decrease the viability of acute leukemia cells. 0.05 x 10^6^ K562 cells/ml were cultured for 24 h, 48 h or 72 h with the indicated concentrations of Dynasore, Dyngo-4a or MitMAB. Viability was assessed with PrestoBlue and was normalized to untreated control (0 μM) groups. Data are given as mean + SEM. Two-way ANOVA was used for statistical testing with Dunnett’s posthoc test in order to compare each treated group with the control at each time point.
(TIF)

S1 Movie 0.15 x 10^6^ of CCRF-CEM cells/ml in medium containing 150 nM DRAQ7 and 500 nM CellEvent-caspase-3/7 were added to chamber slides. Images were taken every 10 min. The movie is representative of two independent experiments.
(AVI)

S2 Movie 0.15 x 10^6^ of CCRF-CEM cells/ml in medium containing 150 nM DRAQ7 and 500 nM CellEvent-caspase-3/7 were added to chamber slides. At 0 min, 5 μM MitMAB was added. Images were taken every 10 min. The movie is representative of two independent experiments.
(AVI)

S3 Movie 0.15 x 10^6^ of THP-1 cells/ml in medium containing 150 nM DRAQ7 and 500 nM CellEvent-caspase-3/7 were added to chamber slides. Images were taken every 10 min. The movie is representative of two independent experiments.
(AVI)

S4 Movie 0.15 x 10^6^ of THP-1 cells/ml in medium containing 150 nM DRAQ7 and 500 nM CellEvent-caspase-3/7 were added to chamber slides. At 0 min, 5 μM MitMAB was added. Images were taken every 10 min. The movie is representative of two independent experiments.
(AVI)

## Abbreviations

ALL: Acute lymphoblastic leukemia
AML: Acute myeloid leukemia
DIC: Differential interference contrast
MitMAB: Myristyl trimethyl ammonium bromide
PBMC: Peripheral blood-derived mononuclear cell
T-ALL: T-cell ALL
