# Supplementary figures and images for: Dynamin inhibition causes context-dependent cell death of leukemia and lymphoma cells
Source: PLoS One. 2021 Sep 7;16(9):e0256708. doi: 10.1371/journal.pone.0256708 (PMC8423305; doi:10.1371/journal.pone.0256708)

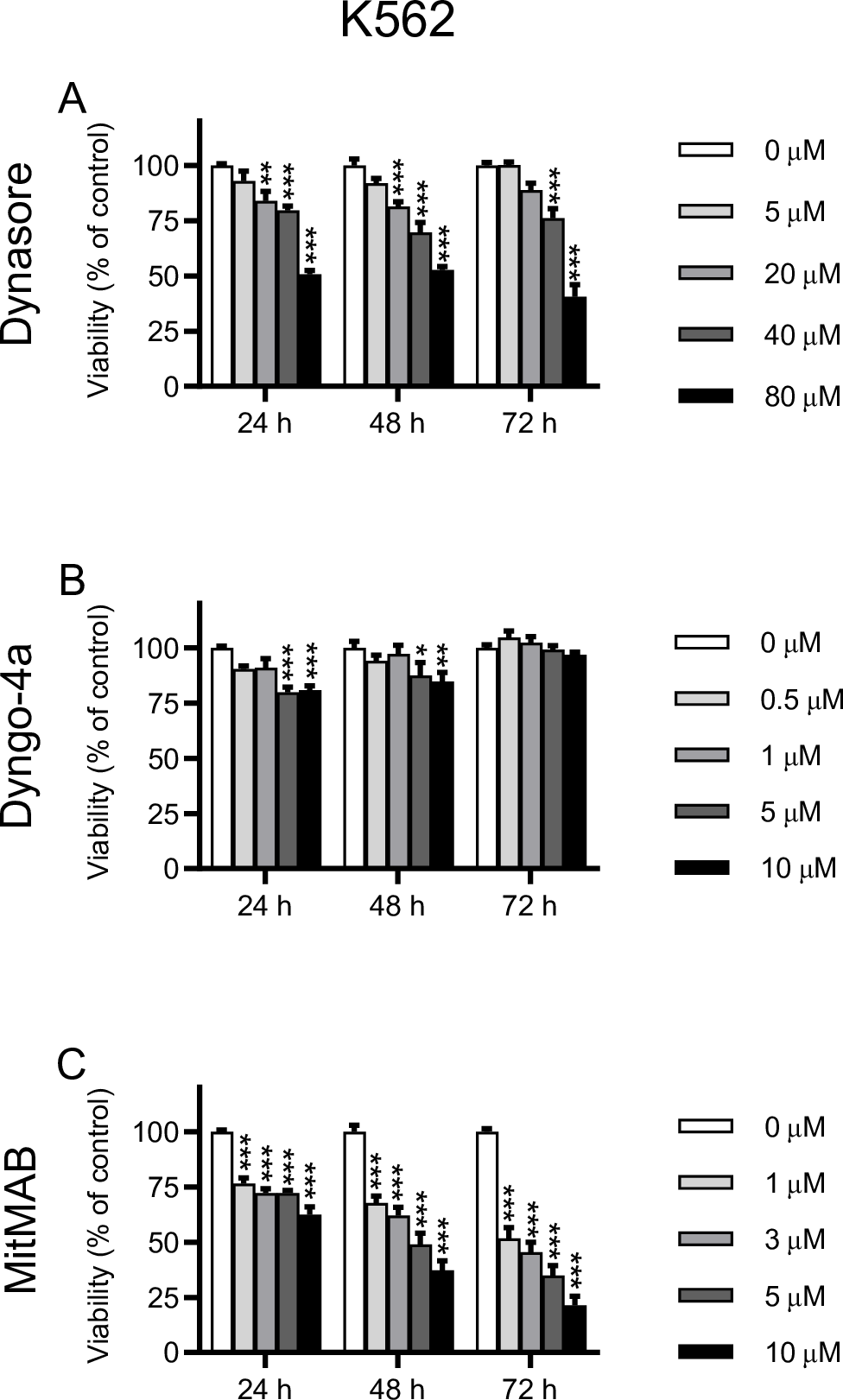

Supplement: S1 Fig — 0.05 x 10^6^ K562 cells/ml were cultured for 24 h, 48 h or 72 h with the indicated concentrations of Dynasore, Dyngo-4a or MitMAB. Viability was assessed with PrestoBlue and was normalized to untreated control (0 μM) groups. Data are given as mean + SEM. Two-way ANOVA was used for statistical testing with Dunnett’s posthoc test in order to compare each treated group with the control at each time point. (TIF) [file pone.0256708.s001.tif]
